# Design of a Linear Wavenumber Spectrometer for Line Scanning Optical Coherence Tomography with 50 mm Focal Length Cylindrical Optics

**DOI:** 10.3390/s22093278

**Published:** 2022-04-25

**Authors:** Sevin Samadi, Masoud Mohazzab, Javad Dargahi, Sivakumar Narayanswamy

**Affiliations:** 1 Department of Mechanical, Industrial and Aerospace Engineering (MIAE), Concordia University, Montreal, QC H3G 1M8, Canada; s_samadi@encs.concordia.ca (S.S.); siva.narayanswamy@concordia.ca (S.N.); 2 Seurat Technologies Inc., 70 Chestnut st, Andover, MA 01810, USA; mmohazzab@seuratech.com

**Keywords:** SD-OCT, spectral analysis, line scanning, linear k space, Grism, cylindrical mirror

## Abstract

Optical coherence tomography (OCT) has a wide range of uses in bioimaging and nondestructive testing. Larger bandwidth light sources have recently been implemented to enhance measurement resolution. Increased bandwidth has a negative impact on spectral nonlinearity in k space, notably in the case of spectral domain OCT (SD-OCT). This nonlinearity reduces the depth-dependent signal sensitivity of the spectrometers. A grating and prism combination is extensively used for linearizing. In an earlier study, we used a combination of the reflective grating and prism, as well as a cylindrical mirror with a radius of 180 mm, to achieve a high SR ratio with low nonlinearity. A creative design for a spectrometer with a cylindrical mirror of radius 50 mm, a light source with a center wavelength of 830 ± 100 nm (μm^−1^ − 6.756 μm^−1^ in k-space), and a grating of 1600 lines/mm is presented in this work. The design optimization is performed using MATLAB and ZEMAX. In the proposed design, the nonlinearity error reduced from 157${}^{\circ }\times$ μm to 10.75${}^{\circ }\times$ μm within the wavenumber range considered. The sensitivity research revealed that, with the new design, the SR ratio is extremely sensitive to the imaging optics’ angles. To resolve this, a spectrometer based on Grism is introduced. We present a Grism-based spectrometer with an optimized SR ratio of 0.97 and nonlinearity of 0.792${}^{\circ }\times$ μm (Δθ/Δk). According to the sensitivity study, the Grism-based spectrometer is more robust.

## 1. Introduction

Optical coherent tomography (OCT) has received a lot of interest recently because of its potential use in non-destructive tissue imaging and industrial component testing (OCT). A wide-band light source with poor coherence is used in OCT, which is an interferometry method [1]. OCT can detect backscattered light from tissues with lateral and depth resolutions in the μm range, two orders of magnitude finer than ultrasound [2]. Time-domain interference is the basis for the majority of OCT equipment; however, because of its great sensitivity and speed, spectral-domain optical coherence tomography (SD-OCT) has attracted a lot of attention lately [3,4,5,6,7,8,9]. By using spectrometers to evaluate the scattered light from the sample’s layers instead of moving the reference mirror, SD-OCT eliminates the requirement for moving the sample itself. An ideal spectrometer consists of a diffraction grating, fold mirrors, and a focusing optical system. The grating’s diffracted angle is directly related to its wavelength. However, with SD-OCT, resampling from wavelength to wavenumber space is required for the Fourier Transformation of output findings. Inversely related to wavenumber (k=2π/λ), the diffraction angle of light from the grating is nonlinear. There are two concerns with this nonlinearity. To begin, the axial point spread function will be increased in scope. Another factor affecting depth sensitivity is the nonuniformity of the wavenumber bandwidth that is integrated by each pixel in a display [10,11]. It is also possible to reduce the nonlinearity by creating specialized hardware configurations that sample the fringe signal directly and in linear form. A prism-based spectrometer for SD-OCT has been suggested [12]. In addition, a k-mapping function may be used to acquire and resample the nonlinear K space signal in post processing to a linear k representation [13].

An additional advantage of the k-mapping technique is that it allows the nonlinear K space signal to be sampled and then converted back to the linear k form during post-processing. Increasing the amount of pixels may also help to counteract this loss of sensitivity [11,14,15]. More pixels in the same region decrease the pixel size, which results in a lot of distortion because of the smaller pixels. The scattered spectrum’s point spread function (PSF) will be greater than the spectrometer’s pixel size in this situation, limiting its performance [15,16,17]. As the number of pixels rises, so do the system costs and processing times, both of which are increasing exponentially. For better signal sensitivity, researchers developed the linear k-space spectrometer. The nonlinearity of the grating is compensated by using a prism to linearize the spectrometer [7,15,16]. Grisms [18] may be created by placing a diffraction grating in front of a prism with an optimal angle between them. Signal sensitivity has been significantly improved by linearizing the k space spectrometer in these studies [4,5]. Light sources employed in these studies have a bandwidth of 40 to 70 nm [4,14,16,17], despite the fact that linearity has improved significantly. This is not the case, however, when it comes to ultra-broadband OCT systems that can identify finer details [5,19,20]. An increase in the spectral bandwidth of the source enhances the OCT’s axial resolution.

Additionally, resolving concerns with picture quality must be dealt with after linearizing the broadband source. As far as we know, chromatic aberration plays an important role in lens-based focusing systems [16,21]. Additional picture quality degradation is caused by motion artifacts [16] in current state-of-the-art scanning, which is fly spot scanning [22]. Line-scanning OCT using cylindrical mirrors was offered as a solution to picture quality difficulties caused by lens focusing and fly spot scanning [22,23,24]. Since 2016, there has been concentrated work on line field OCT from Liverpool university [25,26]. A novel spectrometer was suggested in our prior paper to solve chromatic aberration and depth sensitivity fall-off [15,24,27,28]. A refractive grating and a prism were used to linearize the output. The linearity issue was resolved; however, the SR ratio dropped significantly as a result of that design. A prism and a reflecting grating were then employed to linearize the output of the laser. The spectrometer’s size is increased by using a cylindrical mirror with a 180 mm radius [15]. The cylindrical mirror of the spectrometer is shrunk from 180 mm to 50 mm in this study. We’ve discussed this before. Using this spectrometer, you can obtain data from a line width of 2 mm × 40 μm. The primary contribution of this study is the design and optimization of the line space spectrometer to obtain equidistant output based on a wavenumber equal to one. In comparison to post-Fourier transform systems, the linearity of the resultant spectrum on the image sensor results in better resolution. This work assumes significance with the recent development of Line field OCT systems by the Liverpool University together with the Oxford Instruments [29].

This paper presents a novel line-based spectrometer for SD-OCT that utilizes a cylindrical mirror with a radius of 50 mm. By decreasing the radius of the concave mirror, more light is concentrated on the prism. It is necessary to include an additional mirror in the design in order to better direct the light. This new design has been subjected to a sensitivity analysis, and the results reveal a wide range in the SR ratio. There is a sensitivity analysis included in the publication. This work presents a fresh design using Grism in order to lessen the sensitivity. The first time a k-space spectrometer has been linearized using Grism for line scanning SD-OCT, to the best of our knowledge. In addition to being commercially accessible, Grism decreases the design complexity significantly. In order to develop and improve the spectrometers, we employ ZEMAX Optic Studio software and MATLAB. Optimizing the spectrometer’s design parameters reduced nonlinearity and improved picture quality. Images are judged on their Strehl ratio (SR). ZEMAX calculates the Strehl ratio by measuring the ratio of point spread function (PSF) of an optical system over PSF of an ideal system (without aberrations) and is a measure of how close the system is to an ideal diffraction limited optical system. Zemax does the calculation for users and even it can be used for optimization of the performance of the system. The SR of an imaging system must be more than or equal to 0.8 according to the Marechal criteria. Comparing the spectrometer’s performance against transmission and reflective grating systems using 180 mm cylindrical lenses for focusing, researchers found that it outperforms them both [15]. Table 1 summarizes the SR ratio and nonlinearity errors comparing various designs.

## 2. Theory and Properties

As shown in Equation (1), the exit angle of a reflective grating is proportional to the angle of the input light and its wavelength.

(1) $$d[\sin\left(\theta_{i}\right)+\sin\left(\theta_{m}\right)]=m\lambda$$

where *d* is the grating period spacing, $\theta_{m}$ is the diffraction angle, $\theta_{i}$ is the incident angle, *m* is the diffraction order, and λ is the wavelength.

**Figure 1:**
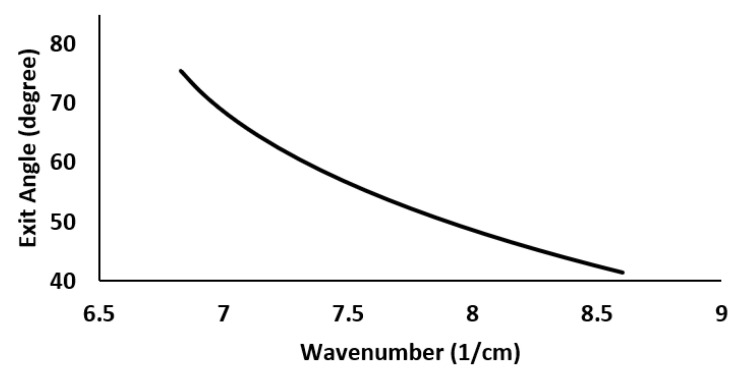
Exit angle of the light from the grating vs. wavenumber.

The nonlinear connection between wavenumber and diffraction angle is illustrated in Figure 1 for a reflective grating with 1600 lines/mm, ‘m’ of 1 and ‘λ’ which varies between 730–930 nm [μm^−1^− 6.756 μm^−1^ in k-space] for the reflective grating.

As seen in Figure 1, the angle between equal separation wavenumbers in reflecting gratings is not linear. Dispersion prisms, on the other hand, order wavelengths in the opposite direction than diffraction gratins. One would infer that, by combining a grating and a prism, a linear relationship between the dispersed wavenumbers and their angles can be obtained.

**Figure 2:**
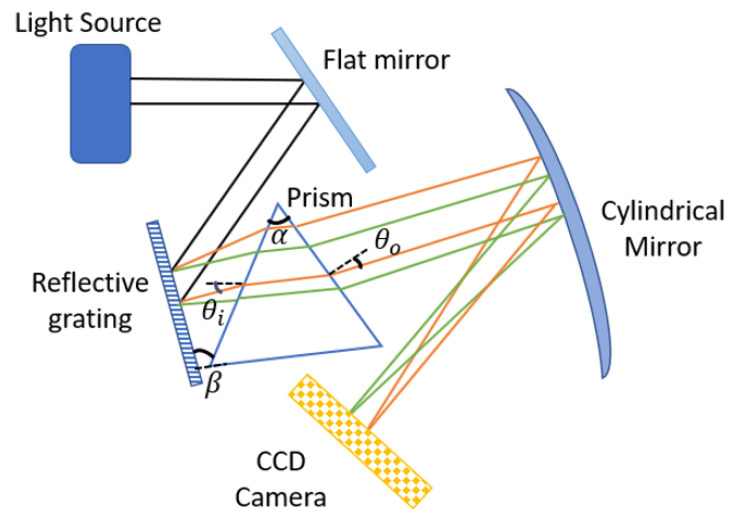
Schematic of the spectrometer using reflective grating and prism (two wavelengths, orange shorter and green longer).

Additionally, this linearity improves depth profile precision by balancing the wavenumber response of such devices in OCT. To ensure linearity, a prism is placed at a predetermined angle (beta) between the grating and the focusing mirror, as indicated in Figure 2. The wavenumbers are incident at a sharp angle on the grating in this arrangement. The angle (beta) was optimally set to ensure that the prism’s center wavelength propagates parallel to the prism’s base. This results in a reduction of the wavelength’s angular variation on either side of the center wavelength, achieving linearity [17].

The dispersion formula for angular wavelength dispersion can be expressed as

(2) $$\theta_{i}\left(\lambda \right)=\arcsin\left(n\left(\lambda_{c}\right)\cos\left(\alpha \right)\right)+\arcsin\left(d\lambda -0.5d\lambda_{c}\right)-\arcsin\left(0.5d\lambda_{c}\right)$$

(3) $$\theta_{o}=\arcsin\left(n_{k}\left(\sin\left(\alpha -\arcsin\left(\frac{\sin(\theta_{k})-\beta }{n_{k}}\right)\right)\right)\right)$$

in which *d* is the grating period spacing, *n* is the wavelength-dependent refractive index of the prism, and $n_{k}$ is the wavenumber-dependent refractive index of the prism.

$\theta_{i}$ is the incident angle to the prism, $\theta_{o}$ is prism’s dispersion angle, $\theta_{k}$ is the diffracted angle of each wavenumber, $\sin\theta_{k}=2\pi ⁄kd-\sin\theta_{i}$. α is the apex angle of the prism, and $\lambda_{c}$ is the center wavelength of the light source.

**Figure 3:**
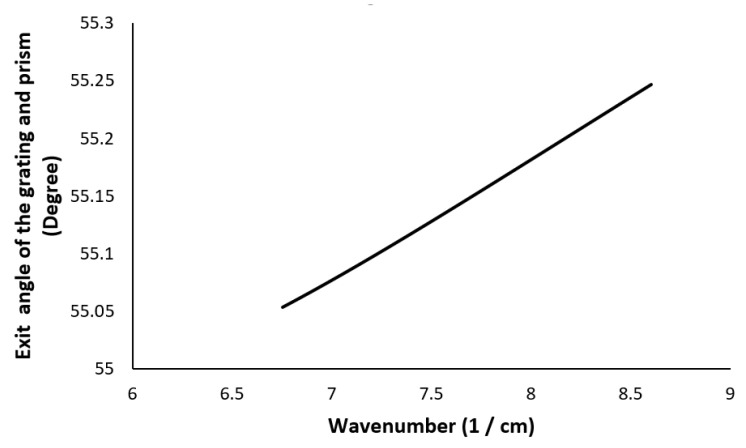
The angular variation of the exit angle for reflective grating and prism combination.

The value of α is set to 60∘, and the value of *d* is set to 1600 lines/mm. Each prism material has a unique value for β when α and d are specified. The $\theta_{o}$ is the function of k. Its variation should be minimal across different wavenumbers (3). The smallest angular variation assures that the $\theta_{o}$ remains linear in k-space. The *x*-axis plots the wavenumber range, while the *y*-axis plots the exit angle. In the illustration, the prism is built of F2, and the angle between it and the grating (β) is $28^{\circ }$. The departure angle changes by 0.2^∘^, as indicated in Figure 3. In comparison, over the same wavenumber range, the exit angle variation for a reflective grating without a prism is $32^{\circ }$ (Figure 1).

With α of 60${}^{\circ }$, β of 28${}^{\circ }$ and d of 1600 lines/mm, the nonlinearity error (Δθ/Δk) is reduced from 157${}^{\circ }\times$ μm in case of grating, to 10.27${}^{\circ }\times$ μm while using the combination of the prism and grating for the wavenumber range considered.

In our earlier publication [27], we designed all reflective spectrometer with SR ratio in range of 0.96–0.97 for all wavenumbers considered. Subsequently, we linearized that design with a prism and a cylindrical mirror of 180 mm radius was used for focusing [15]. In this work, our goal is to miniaturize the spectrometer with a 50 mm cylindrical mirror for focusing. However, size reduction imposes significant barriers to optimizing beam quality.

## 3. Spectrometer with Reflective Grating and Prism

**Figure 4:**
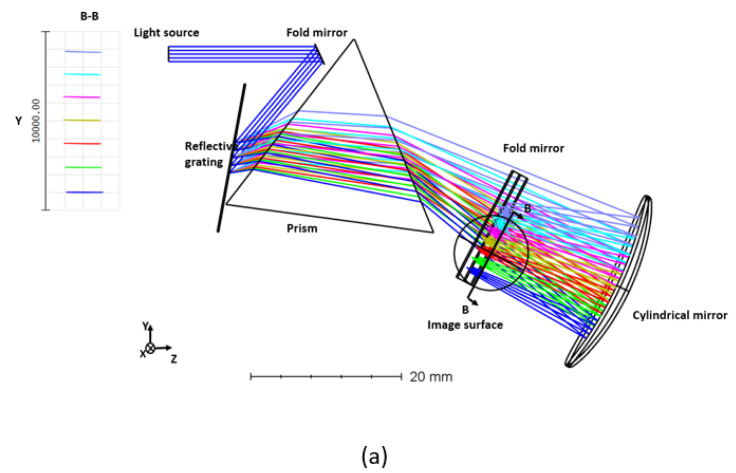

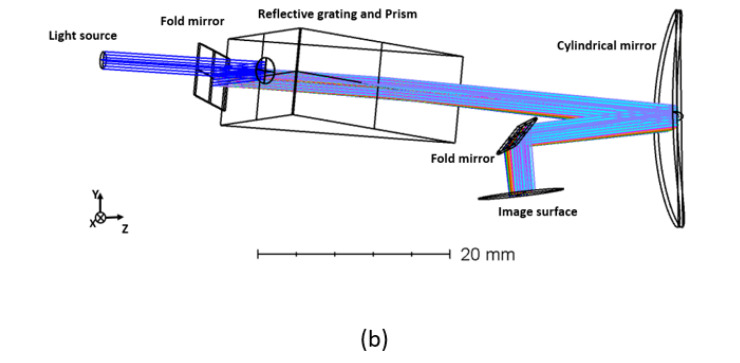
Design of a spectrometer with a prism and reflective grating (**a**) side view; (**b**) top view.

In ZEMAX, a novel spectrometer with reflective grating and prism was developed to determine the linearity of the exit angle, as illustrated in Figure 4. A fold mirror, reflecting grating, and a cylindrical focusing mirror comprise the structure of this spectrometer. This design considers a superluminescent laser diode (SLD) source with a central wavelength of 830 nm and a bandwidth of 200 nm (8.607 μm^−1^− 6.756 μm^−1^). The broadband spectrum is dispersed using a combination of reflective grating (1600 lines/mm) and Prism (F2 material with an apex angle α of $60^{\circ }$). Following the grating and prism combination, the light is focused onto the detector using a concave cylindrical focusing mirror.

**Figure 5:**
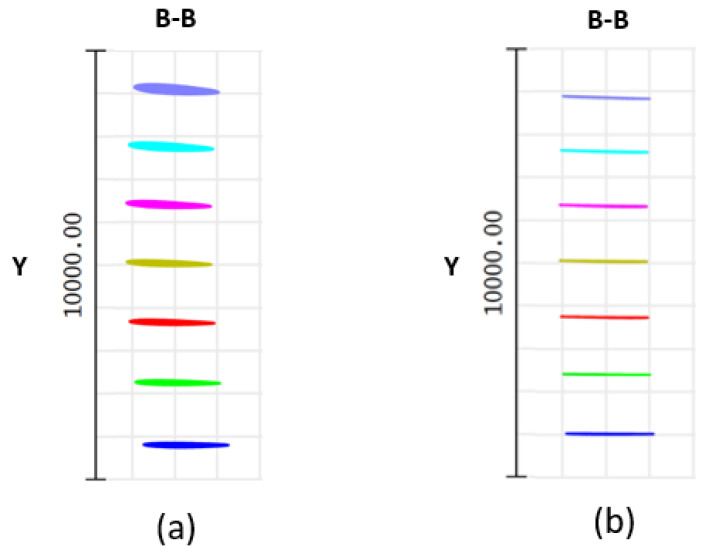
(**a**) Spot diagram before optimization; (**b**) spot diagram after optimization.

The optical elements are initially spaced as follows: the distance between the fold mirror and the grating is 19 mm, the distance between the grating and the prism is 23 mm, and the radius of the focusing mirror is 50 mm. The challenge with a smaller focal length mirror is that the light reflected focuses very close to the mirror. Placing a detector at the focal plane interferes with the incoming light. Moreover, improved beam quality results when the beam incidents the cylindrical mirror with the highest symmetry. This restricts the angle of the cylindrical mirror. To address this, a fold mirror (tilted $45^{\circ }$) is located 18 mm from the cylindrical mirror. The output of the PSF is illustrated in Figure 5a. While the output is linear, the scanned lines are not parallel to the *x*-axis. In addition, the beam quality at the focal plane progressively worsens with decreasing wavenumber. Subsequently, using the merit functions, the angles and distances are optimized to reduce image plane aberration and linearize the output. After optimization, the distance between the fold mirror and the grating is 18 mm, the distance between the grating and the prism is 22.5 mm, and a fold mirror (tilted $45^{\circ }$) is located 18.5 mm from the cylindrical mirror. The PSF output of optimized design is shown in Figure 5b.

### Spectrometer Analysis

**Figure 6:**
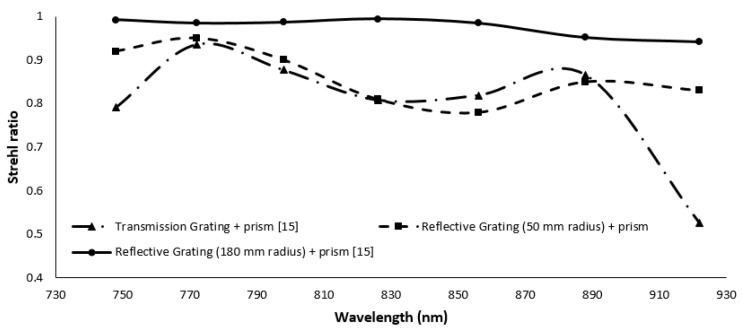
Strehl ratio of the proposed spectrometer and previous works [15].

With the prism and grating combination, the nonlinearity error is reduced from 157${}^{\circ }\times$ μm (Δθ⁄Δk) to 10.27${}^{\circ }\times$ μm (Δθ⁄Δk). MATLAB simulation showed the same nonlinearity errors as ZEMAX. The SR of different wavelengths in the proposed design is shown in Figure 6. From the figure, it can be seen that the SR ratio for all the wavelengths in combination with the reflective grating and prism varies between 0.8–0.96. In addition, in the figure, the SR ratio of the designs reported in our earlier work [15] are shown in a broken line with triangular markers and a solid line. The SR for some of the wavelengths in the case of transmission grating and prism combination is lower than the Marechal criterion.

**Figure 7:**
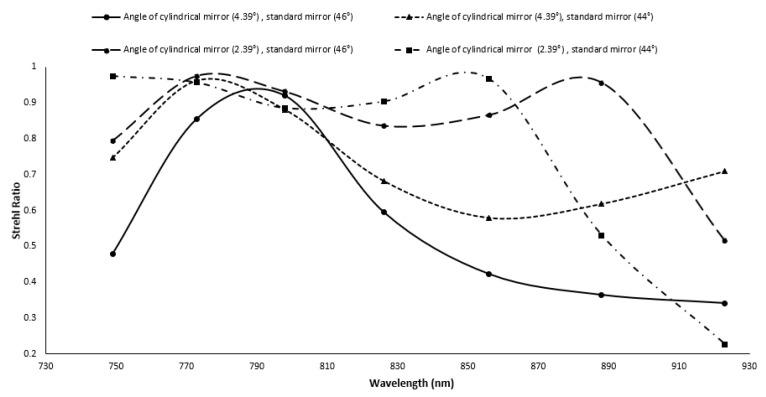
Strehl ratio of the proposed spectrometer for different angles of the optical components.

**Figure 8:**
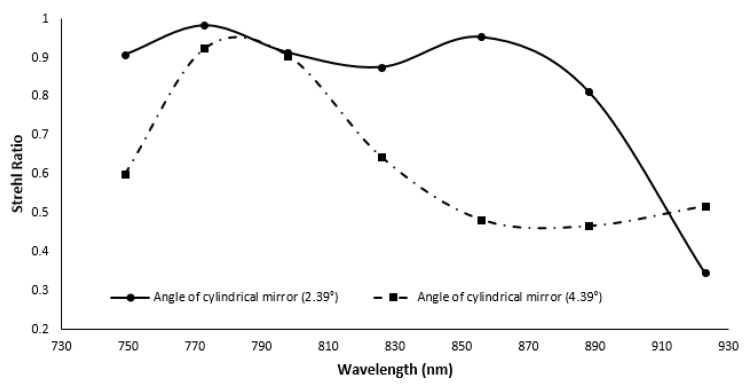
Strehl ratio of the proposed spectrometer for variation of the cylindrical mirror’s angle.

While the reflecting grating and prism have a lower nonlinearity error (0.0149^∘^ × μm), the reflective grating system has a higher SR ratio. The nonlinearity error of the reflective grating and prism increased by decreasing the radius of the cylindrical mirror from 0.03517^∘^ × μm to 10.75^∘^ × μm. Additionally, when the radius is lowered to 50 mm, the SR ratio decreases, but it still meets the Marechal criterion. The SR ratio of the spectrometer is highly sensitive to the angle of the cylindrical mirror and fold mirror used in the imaging. To determine the robustness of the design, the angle of the cylindrical mirror is varied from $3.39\pm 1^{\circ }$ and the angle of the fold mirror is varied from $45\pm 1^{\circ }$ in ZEMAX. The SR ratio for different wavelengths within the range are recorded for different combinations. The results are summarized in Figure 7. From the figure, it can be seen that the SR ratio is highly sensitive to the angle, and it can vary from 0.9–0.2 within the given wavelength range for $\pm 1^{\circ }$ variation of the cylindrical and fold mirror. If the fold mirror was kept constant and only the cylindrical mirror was moved, the SR still drops from 0.9 to 0.3 for the given wavelength range. From the sensitivity analysis, it is clear that the nonlinearity is not affected by the alignment, but the SR ratio is highly sensitive to the angles of the optical components (Figure 8).

There is a sensitivity analysis of the prism’s exit angle in relation to the grating and prism angle (β) in our publication [15]. Using the transmission grating, the exit angle was not affected by β.

From the sensitivity analysis, we know that the design is extremely sensitive to the angles of the concave mirror and fold mirror. On the other hand, the system is robust with respect to the variations in β. Reducing β from 28${}^{\circ }$ to 0${}^{\circ }$ will not only enable a simpler alignment process but will also not affect the nonlinearity. However, it is not possible to have a reflective grating with prism together at this angle (β = 0). Transmission grating and prism combination at β = 0 is essentially a Grism based system. To develop a robust system, a new Grism based spectrometer will be discussed.

## 4. Spectrometer with Grism

**Figure 9:**
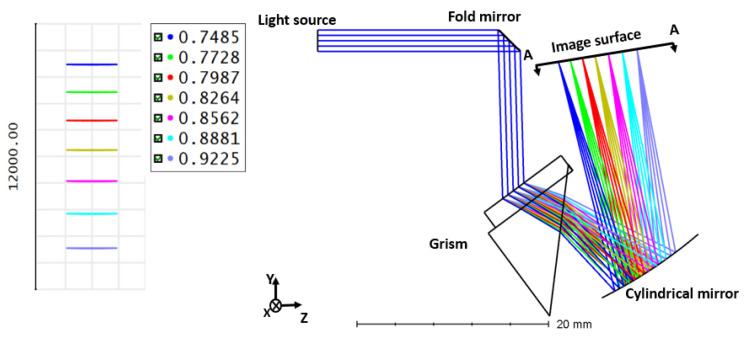
Design of a spectrometer with Grism [30].

Design of the linear k space spectrometer with Grism as a dispersing component is depicted in Figure 9. The components of the Grism based spectrometer include a fold mirror, a Grism, and a concave cylindrical mirror and an imaging sensor. The interfering light is reflected 15 mm distant from the fold mirror in the direction of the Grism. In relation to the grating, the beam angle is 60${}^{\circ }$. The grating (d = 1600 lines/mm) and right-angle prism (F2 material) are coupled to make a prism with a 45${}^{\circ }$ apex angle.

The scattered beam is imaged onto a detection surface by a cylindrical concave mirror with a radius of 50 mm. The inset in Figure 9 illustrates the dispersion on the image surface (footprint diagram) at equal wavenumber spacing [30].

### Spectrometer Analysis

**Figure 10:**
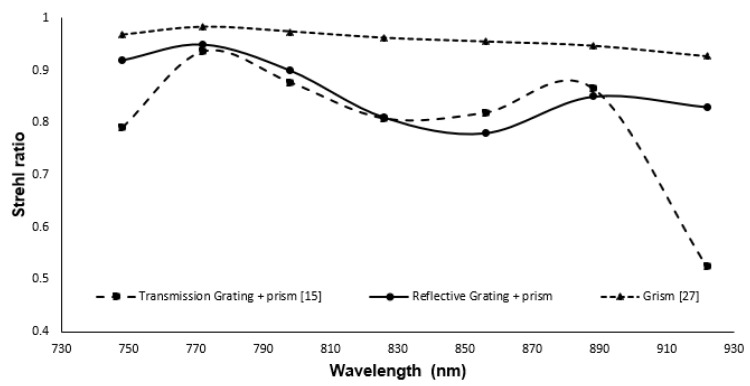
Strehl ratio of different spectrometers designed with reflective grating and prism, transmission grating and prism and Grism.

The result from the footprint diagram shows a nonlinearity error of 0.792${}^{\circ }\times$ μm (Δθ/Δk) within the wavenumber range. This is a significant improvement from the nonlinearity error of 10.75${}^{\circ }\times$ μm in our earlier design. Moreover, the Strehl ratio for different wavelengths on the focal plane with Grism is better than our earlier design with reflective grating and prism. Figure 10 shows the optical performance of the Grism (broken line with triangular pointers) in comparison with that of the reflection grating and prism combination (line with circular pointers). There will be background noise due to reflection between transmission grating and prism; however, the optical surfaces will be coated with anti-reflection coating at proper wavelength range for noise reduction [31,32].

The optical performance of the transmission grating and prism combination (broken line with square pointers) is also shown in the figure for [15].

**Figure 11:**
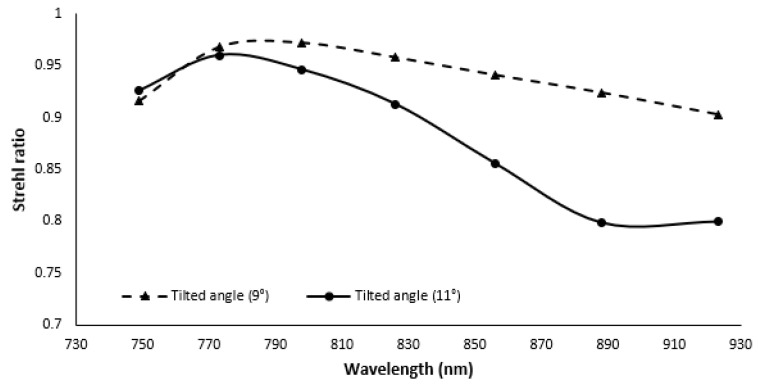
Strehl ratio of the proposed spectrometer for variation of cylindrical mirror angle.

To test the robustness of the Grism based spectrometer, sensitivity analysis of the imaging optics is performed, and the results are shown in Figure 11. From the figure, it is clear that, when the concave mirror position was changed by −1${}^{\circ }$ with respect to the optimal position, the SR remains above 0.9 for all the wavelengths. On the other hand, when the concave mirror position was changed by +1${}^{\circ }$ with respect to the optimal position, the variation in SR is higher, but the lowest SR still remains above the Marechal criterion. Compared to the sensitivity analysis of the reflective grating and prism, the Grism based spectrometer is more robust.

**Figure 12:**
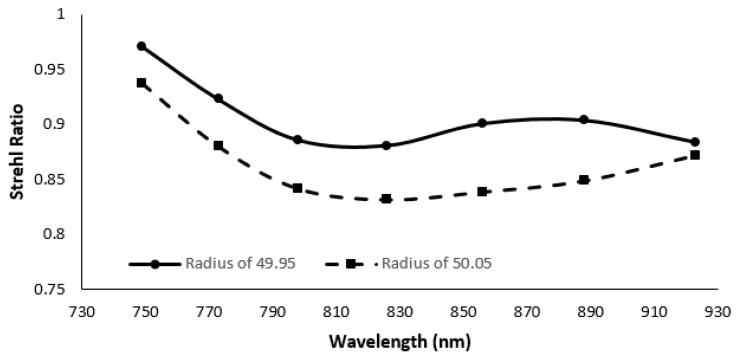
Strehl ratio of the proposed spectrometer for variation of cylindrical mirror’s radius.

**Figure 13:**
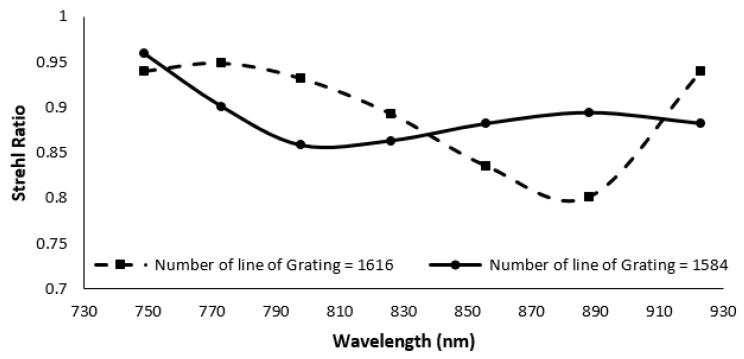
Strehl ratio of the proposed spectrometer for variation of number of lines of grating.

The robustness of the Grism based spectrometer to the precisions in fabrications of optical components like concave mirror radius and number of lines of grating is analyzed. From the SR analysis, it is clear that, when the concave mirror radius was changed to 50.05 mm, the variation of the SR remains above 0.9 for all the wavelengths. On the other hand, when the concave mirror radius was changed to 49.95 mm, the variation in SR is higher, but the lowest SR still remains above the Marechal criterion. In addition, by changing the number of lines of the grating to 1600 ± 0.016, we checked the robustness of the spectrometer. SR remained above Marechal criterion for both designs. The results are shown in Figure 12 and Figure 13.

## 5. Comparison of All the Spectrometers

The optical performance of spectrometer for OCT is a combination of the nonlinearity error and the SR ratio. The performance metrics of the linear k space spectrometers designed in this work are shown in Table 1. For comparison, the output from spectrometers with either transmission or reflective grating without a prism is also shown. From the table, it can be seen that the nonlinearity error is least for transmission grating and prism combination; however, the SR varies between 0.55–0.98. On the other hand, for the combination of the reflective grating and prism with a 180 mm concave mirror, the SR is high and the nonlinearity error is low. Compared to all other spectrometers, this has a very large concave mirror, thereby increasing its size. The results are shown in Table 1.

The SR is calculated to show the robustness of the designed spectrometer for errors in fabrications of optical components. The results are indicated in Table 2.

**Figure 14:**
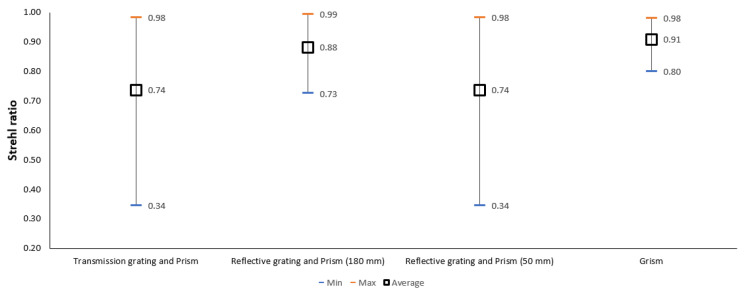
Strehl ratio of different spectrometers.

The miniaturized version with a 50 mm radius concave mirror provides an SR ratio above the Marechal criterion and lesser nonlinearity error. However, the optical quality of the system is extremely sensitive to variation of the angles of the optics. To compare the robustness of the linear K space spectrometer designs, a sensitivity analysis is performed and the results shown in Figure 14.

In Figure 14, the *x*-axis shows the different linear k space spectrometers designed as part of this work, and the *y*-axis shows the imaging quality as the SR ratio. From the graph, it can be seen that the best SR ratio with the least variance is possible with Grism. While the nonlinearity is the least with a combination of transmission grating and prism, this design has the lowest imaging quality and the least robustness. On the other hand, the imaging quality is very high on reflective grating with a prism (180 mm) and the nonlinearity error is significantly lower. However, the size of the system is larger and the design is not robust as the imaging quality reduces below the Marechal criterion as shown in Figure 14. Considering these, the spectrometer designed with Grism is the ideal spectrometer for SD-OCT applications with a nonlinearity of 0.792 and the SR ratio above 0.97 for all wavelengths. From Figure 14, it can be seen that the Grism is the least sensitive to variation in optical angles with the SR ratio always being above the Marechal criterion.

## 6. Conclusions

For line scanning SD-OCT, this paper presents two different designs of linear wavenumber spectrometers using cylindrical optics. For linearizing, a reflective grating and prism combination are used. To acquire the angles of the prism and grating, a MATLAB analysis is performed initially, followed by a ZEMAX simulation. The main contribution is to minimize the cylindrical mirrors radius to 50 mm so that the spectrometer footprint remains smaller while imaging 2 mm × 2 mm × 2 mm. From the sensitivity analysis, it can be concluded that the SR ratio reduced to 0.2 (far below the Marechal criterion) even for smaller variations ($\pm 1^{\circ }$) in the alignment of critical optics. To overcome this, a Grism as the dispersion group is proposed. The Grism based spectrometer design was optimized for nonlinearity and high SR ratio. The nonlinearity error is reduced from 10.75${}^{\circ }\times$ μm in the case of reflective grating and prism, to 0.79${}^{\circ }\times$ μm, while using the Grism. Reducing the nonlinearity error can lead to high axial resolution and reduce the signal sensitivity fall off. The SR ratio for the Grism based spectrometer is found to be 0.97–0.98, and the spectrometer is found to be robust.

## Figures and Tables

**Table 1:** Summary of the designed spectrometers.

Spectrometer	Wavelength (nm)	Nonlinearity Error (Δ*θ*/Δ*k*)	SR Ratio
Transmission grating [15]	730–930	147.0115	0.98–0.996
Reflective grating [27]	730–930	157	0.96–0.97
Transmission grating + prism [15]	730–930	0.0149	0.55–0.98
Reflective grating (180 mm radius) + prism [15]	730–930	0.03517	0.95–0.98
Reflective grating (50 mm radius) + prism	730–930	10.75	0.8–0.96
Grism	730–930	0.792	0.97–0.98

**Table 2:** Strehl ratio for precisions in practical fabrications of optical components.

Spectometer	Wavelength (nm)	SR Ratio
Grism (*d* = 1616)	730–930	0.801–0.949
Grism (*d* = 1584)	730–930	0.858–0.959
Grism ( 50.05 mm radius)	730–930	0.832–0.937
Grism (49.95 mm radius)	730–930	0.881–0.971

## Data Availability

Not applicable.
